# Microbial electroactive biofilms dominated by *Geoalkalibacter* spp. from a highly saline–alkaline environment

**DOI:** 10.1038/s41522-020-00147-7

**Published:** 2020-10-13

**Authors:** Sukrampal Yadav, Sunil A. Patil

**Affiliations:** Department of Earth and Environmental Sciences, Indian Institute of Science Education and Research Mohali (IISER Mohali), Knowledge City, Sector 81, SAS Nagar, Punjab, 140306 India

**Keywords:** Biofilms, Metagenomics, Microbial ecology, Applied microbiology, Water microbiology

## Abstract

Understanding of the extreme microorganisms that possess extracellular electron transfer (EET) capabilities is pivotal to advance electromicrobiology discipline and to develop niche-specific microbial electrochemistry-driven biotechnologies. Here, we report on the microbial electroactive biofilms (EABs) possessing the outward EET capabilities from a haloalkaline environment of the Lonar lake. We used the electrochemical cultivation approach to enrich haloalkaliphilic EABs under 9.5 pH and 20 g/L salinity conditions. The electrodes controlled at 0.2 V vs. Ag/AgCl yielded the best-performing biofilms in terms of maximum bioelectrocatalytic current densities of 548 ± 23 and 437 ± 17 µA/cm^2^ with acetate and lactate substrates, respectively. Electrochemical characterization of biofilms revealed the presence of two putative redox-active moieties with the mean formal potentials of 0.183 and 0.333 V vs. Ag/AgCl, which represent the highest values reported to date for the EABs. 16S-rRNA amplicon sequencing of EABs revealed the dominance of unknown *Geoalkalibacter* sp. at ~80% abundance. Further investigations on the haloalkaliphilic EABs possessing EET components with high formal potentials might offer interesting research prospects in electromicrobiology.

## Introduction

Electromicrobiology is a new subdiscipline of (environmental) microbiology, which deals with the study of electrochemical interactions or extracellular electron transfer (EET) processes between microorganisms and the solid-state electron acceptors or donors, and their implications in different environments^1,2^. The microorganisms which use EET to achieve their respiratory or metabolic processes are referred to as electroactive microorganisms (EAMs)^3,4^. These are further subcategorized into exoelectrogens and electrotrophs. Exoelectrogens use outward EET to reduce the extracellular solid-state electron acceptors, such as mineral oxides and electrodes, and achieve their respiration. In contrast, electrotrophs use inward EET to oxidize the solid-state electron donor sources, in order to maintain their respiratory and metabolic activities. These microorganisms play important roles in different biogeochemical processes, such as mineral recycling^5–7^ and interspecies electron transfer^8,9^, and are used for the development of various applications ranging from wastewater treatment and concomitant water reclamation and energy production^10,11^, bioproduction to bioremediation, and biosensing^1,12^.

Strengthening of the foundation of electromicrobiology as a major discipline in microbiology requires a broader and improved understanding of EAMs and their EET mechanisms from different environments. In particular, understanding the diversity of extreme EAMs with different metabolic capabilities holds great potential not only to unravel their ecological significance, but also to the future development of niche-specific microbial electrochemistry-driven biotechnological applications. For instance, the use of extreme EAMs is desired to overcome the limitations associated with sluggish reaction kinetics and electron transfer, poor electrolyte conductivity and associated ohmic losses, and low organics removal efficiencies in bioelectrochemical systems operated at normal conditions. Some of the promising applications for microorganisms possessing extreme electroactivity include harvesting energy (e.g., electricity or hydrogen) from waste streams with extreme characteristics, developing energy-efficient bioproduction processes, and bioremediation of specific pollutants in extreme environments. Researchers have explored mostly normal habitats for the EAMs so far^4,13^. The extreme environments remain poorly studied for such microorganisms, mainly due to difficulties in sampling and conducting in situ experiments, as well as lack of appropriate enrichment protocols^2,14^. A few pure culture isolates of the extreme EAMs are known or available^4,15,16^. Very few studies have reported on the diversity of EAMs from extreme environments. These include, for instance, highly saline^17–19^, extreme acidic^20^ and alkaline^21^, extreme low^22^ and high temperature^19,23^, high temperature and pressure^24,25^, and deep subsurface^26^ habitats. A combination of some of these extreme conditions also exists in some environments. Examples include saline-alkaline or haloalkaline, high temperature–low pH, and extreme saline–high temperature environments that host those microbes, which are adapted to two different extreme conditions. Exploring such habitats is expected to unravel the unknown microbial diversity and metabolic traits that could broaden the understanding of different ecological niches for EAMs and may offer opportunities for their use under specialized extreme conditions.

The electroactivity of a few microorganisms that can grow under highly saline or alkaline or haloalkaline conditions has been studied with the pure culture isolates of *Geoalkalibacter ferrihydriticus, Geoalkalibacter subterraneus, and Natrialba magadii* available in the culture repositories^13,17,21,27,28^. The adaptation and enrichment of the mixed microbial community to develop tolerance to free ammonia under highly saline and alkaline conditions has also been reported^29^. However, the real haloalkaline habitats have barely been explored for the EAMs. For instance, Kumar et al. enriched a mixed microbial community capable of producing bioelectrocatalytic current generation from the haloalkaline sediments of Texcoco Lake, Mexico, but did not analyze the electroactive community^30^. To the best of our knowledge, no study has been conducted on a detailed understanding of the diversity of EAMs from the haloalkaline habitat thus far.

In this study, we present the electrochemical enrichment and characterization of the exoelectrogenic microorganisms from the extreme haloalkaline environment of Lonar Lake. It is the only haloalkaline hypervelocity impact meteorite Crater Lake formed in basaltic rock in the world (Supplementary Fig. 1). It is known for its high saline (ranging between 5 and 24 g/L) and alkaline (9.5–10 pH) environment^31,32^. The variation in the salinity data in the literature is due to variations in the sampling locations and seasons. It has been the hotspot for geochemists, astrobiologists, ecologists, and microbiologists due to its unique characteristics. The lake system has been well explored for the isolation of different microbial strains and to understand the broad microbial diversity^33–35^, but not for the EAMs. For studying the electromicrobiology of this lake, we used the electrochemical enrichment or cultivation approach. It involves the use of polarized electrodes at different potentials as an analog or proxy to different natural terminal electron acceptor conditions essential to the microbial respiratory activities. It was followed by the detailed characterization of the best-performing enriched haloalkaliphilic microbial EABs via electrochemical, microscopic, and 16S-rRNA amplicon sequencing techniques.

## Results

### Sediment characteristics

The sediment samples obtained during winter and monsoon seasons had almost similar pH, but slightly different salinity levels (Table 1). The low salinity in the monsoon season samples is most likely due to the inflow of freshwater into the lake system. A wide range of salinity values ranging from 5 to 24 g/L has been reported for the Lonar lake sediments^31,34^. It is mainly due to variations in the sampling locations and seasons. In this study, we chose 20 g/L salinity and 9.5 pH based on analyzed data (Table 1) and the literature. The sediment analysis also revealed high chemical oxygen demand (COD) values of up to 526.43 ± 13.4 mg/L, thereby suggesting the productive or eutrophic nature of the lake. The dominant organic acid present in the lake sediments was acetic acid at a concentration of up to 62 mg/L. Various soluble ions, such as SO_4_^2−^, PO_4_^3−^, NH_4_^+^, and NO_3_^−^ that are relevant to support the respiratory activities and growth or metabolic activities of microorganisms, are detected in sediments (Table 1). Elements such as Fe, Mg, Mn, Ca, and Na are also present in the Lonar lake sediments^36,37^. Among these, oxidized Fe and Mn compounds are known electron acceptors that can support the microbial respiratory activities in such anoxic environments.

**Table 1:** Physiochemical characteristics of the sediment samples of the Lonar Lake obtained during winter (January) and monsoon (August) seasons.

S. No.	Parameters	January 2019	August 2019
1.	pH	9.8 ± 0.1	9.6 ± 0.2
2.	Salinity (g/L)	19.2 ± 2.6	14.33 ± 1.0
3.	Conductivity (mS/cm) at 24 °C	30.2 ± 3.7	22.03 ± 1.5
4.	Ammonia (mg/L)	2.6 ± 1.1	2.23 ± 0.1
5.	Phosphate (mg/L)	56.0 ± 1.98	47.23 ± 2.6
6.	COD (mg/L)	442.3 ± 204.7	526.43 ± 13.4
7.	Sulfate (mg/L)	77.73 ± 6.1	62.83 ± 1.2
8.	Nitrate (mg/L)	353.33 ± 182.8	222.43 ± 7.0

### Electrochemical enrichment or cultivation of the electroactive microorganisms

**Fig. 1:**
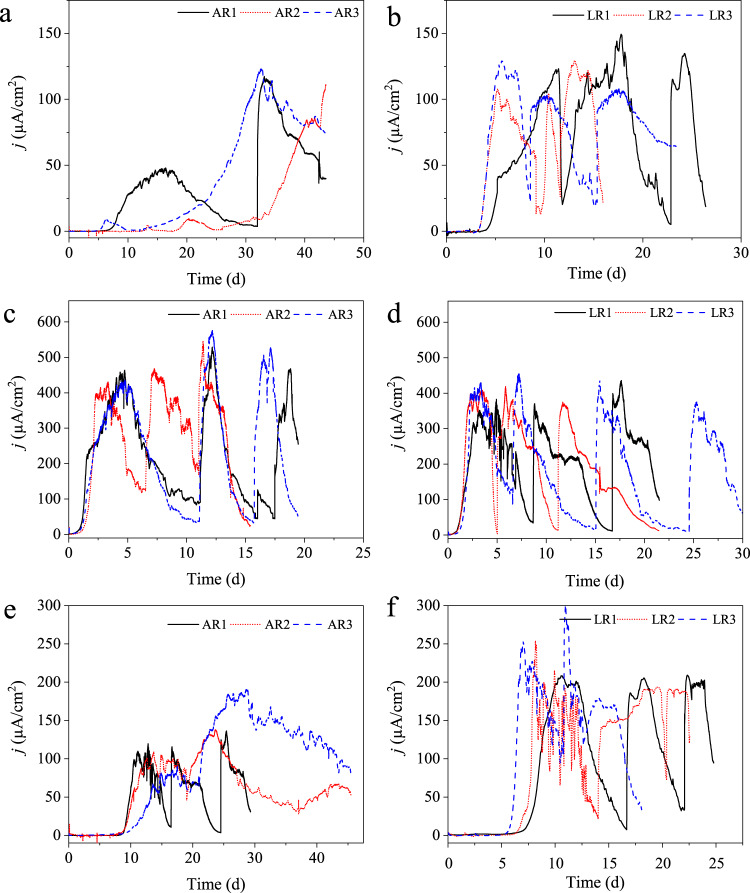
Chronoamperometry profiles at different applied electrode potentials. Bioelectrocatalytic current generation by the enriched microbial EABs at different applied electrode potentials with acetate (**a** 0 V, **c** 0.2 V, and **e** 0.4 V) and lactate (**b** 0 V, **d** 0.2 V, and **f** 0.4 V) substrates.

EAMs can grow by linking substrate oxidation reaction to the reduction of the solid-state electrode in the absence of any other electron acceptor. In such cases, microbial substrate oxidation is directly related to the bioelectrocatalytic current generation, which can be monitored by the chronoamperometry (CA) technique^38^. Figure 1 shows the CA profiles, i.e., bioelectrocatalytic current generation by microorganisms at the electrodes polarized at 0.0, 0.2, and 0.4 V. The reactors with working electrodes polarized at 0.2 V showed current production within 2 days of inoculation (Fig. 1c, d), and achieved the maximum current densities of 548 ± 23 and 437 ± 17 µA/cm^2^ with acetate and lactate substrates, respectively. In the case of 0 and 0.4 V conditions, the start-up time of noticeable current production was >7 days. Moreover, low current densities of 117 ± 6 and 135 ± 12 µA/cm^2^ at 0 V (Fig. 1a, b), and 155 ± 31 and 238 ± 26 µA/cm^2^ at 0.4 V (Fig. 1e, f) were achieved with acetate and lactate, respectively, by the enriched microorganisms compared to 0.2 V condition. The electrodes polarized at −0.2 V showed no or negligible bioelectrocatalytic current response (Supplementary Fig. 2). It is most likely due to a very little difference between the reduction potential of the electron donor (i.e., acetate or lactate) and the potential that was applied at the electrode, which acted as the electron acceptor. Similarly, both controls, namely abiotic connected and biotic unconnected, exhibited no substrate oxidation and current response (Supplementary Fig. 3). Hence, the electric current production in all other experimental conditions can be attributed to the bioelectrocatalytic activity of the enriched haloalkaliphilic microorganisms.

The low bioelectrocatalytic current generation at 0.4 V condition suggests that a high applied electrode potential does not always result in the growth of efficient EAMs, and better bioelectrocatalytic current generation due to most likely less energy harvest^39^. It is in agreement with the published reports on, for example, *Geobacter sulfurreducens* and the mixed-culture-based EABs^40,41^. The maximum bioelectrocatalytic current generation data (Fig. 1) confirm 0.2 V applied potential condition to be the best among all tested potentials for the enrichment of EAMs under the here tested conditions.

On replenishing the spent medium by a fresh medium, the bioelectrocatalytic current response resumed immediately and attained the maximum values within a few days in each batch cycle in all cases (Fig. 1). It suggests that the electroactive microbes attached to the electrodes and growing in the form of biofilm, i.e., electroactive biofilm (EAB) rather than the microbes in the bulk phase were mainly responsible for the bioelectrocatalytic current generation. The coulombic efficiency (CE), i.e., the electrons recovered in electric current, achieved was 52.4 ± 2.3 and 54.2 ± 11% in the case of acetate and lactate-fed biofilms, respectively, grown at 0.2 V. Only slightly >50% electron recovery in electric current might be because of the extra energy requirement by microbes to increase biomass, and to maintain molecular and morphological integrity under such extreme conditions^15,16,42^.

### Electrochemical characterization of the haloalkaliphilic electroactive biofilms

**Fig. 2:**
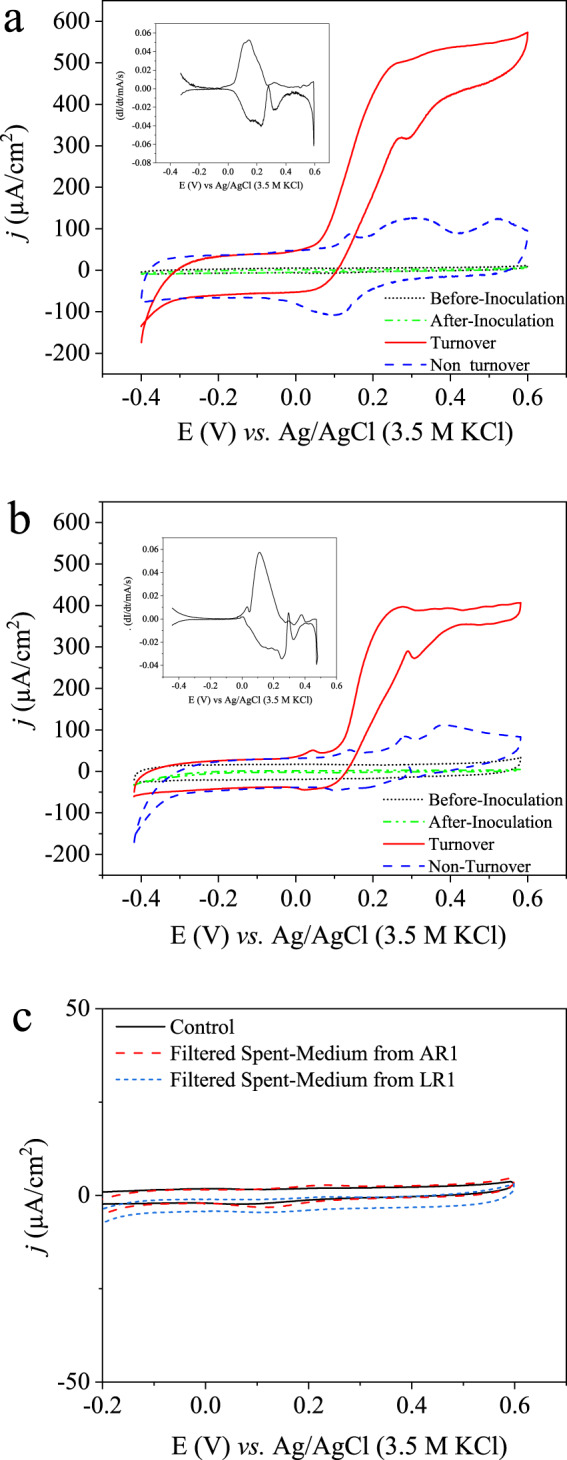
Cyclic voltammograms of the electroactive biofilms enriched at 0.2 V. CVs obtained with a acetate and **b** lactate substrates at different conditions, viz., before and after inoculation, and during substrate turnover and non-turnover. **c** CVs obtained with the new electrodes in a filtered spent medium from the representative acetate- and lactate-fed reactors, and in a fresh electrolyte medium without any substrate and inoculum (control).

Cyclic voltammetry technique was used for this purpose, in which varying potential is applied gradually at the working electrode, and the steady-state current production at each applied potential is recorded^43^. Typical sigmoidal-shaped cyclic voltammogram (CVs) were obtained under the substrate turnover conditions in the case of both acetate and lactate-fed biofilms enriched at 0.2 V (Fig. 2). The first derivative of the turnover CVs of the acetate-fed biofilms revealed two redox-active moieties or components with the formal potentials of ~0.185 and 0.331 V (Fig. 2a). CVs recorded under the non-turnover condition revealed the prevalence of the similar redox peaks. In the case of lactate-fed biofilms also two prominent redox-active moieties with the formal potentials of ~0.182 and 0.335 V were observed (Fig. 2b). No redox peaks were observed in the CVs recorded at two different control conditions, i.e., before (only with growth medium) and immediate after microbial inoculation in the electrolyte medium (Fig. 2a, b). These observations suggest the absence of any soluble redox-active species or mediator at the abiotic electrode surface, in the electrolyte medium and in the microbial inoculum source. The appearance of redox peaks in the CVs recorded under the substrate non-turnover conditions at the end of CA experiments, thus clearly suggests their association with the microbial EAB, and not with the substrate. The midpoint potential of these redox peaks is similar to the ones observed in CVs conducted under the substrate turnover conditions. Other replicate reactors with acetate and lactate substrates showed similar CVs (Supplementary Fig. 5). Based on the applied potential of 0.2 V, it can be inferred that the redox-active moiety with the mean formal potential of 0.183 V was involved EET to the electrode in the case of both acetate- and lactate-fed biofilms. A redox-active moiety with a much higher formal potential than the applied electrode potential is probably not playing any direct role in the electron transfer process in this case. Similar observations have been reported for the *Geobacter-*dominated EABs earlier^44,45^.

Neither any redox peak nor deviation in the faradic current densities was observed in the CVs recorded with the new electrodes in filtered spent media from both acetate- and lactate-fed reactors (Fig. 2c). These observations suggest that no soluble redox-active components or mediators were secreted in the medium by the enriched microbial EAB on the completion of batch experiments, and thereby confirm that the electron transfer and bioelectrocatalytic current generation was due to the electrode-associated biofilms and most likely via direct electron transfer mechanism.

### Visualization of the haloalkaliphilic microbial EABs at the electrode surface

**Fig. 3:**
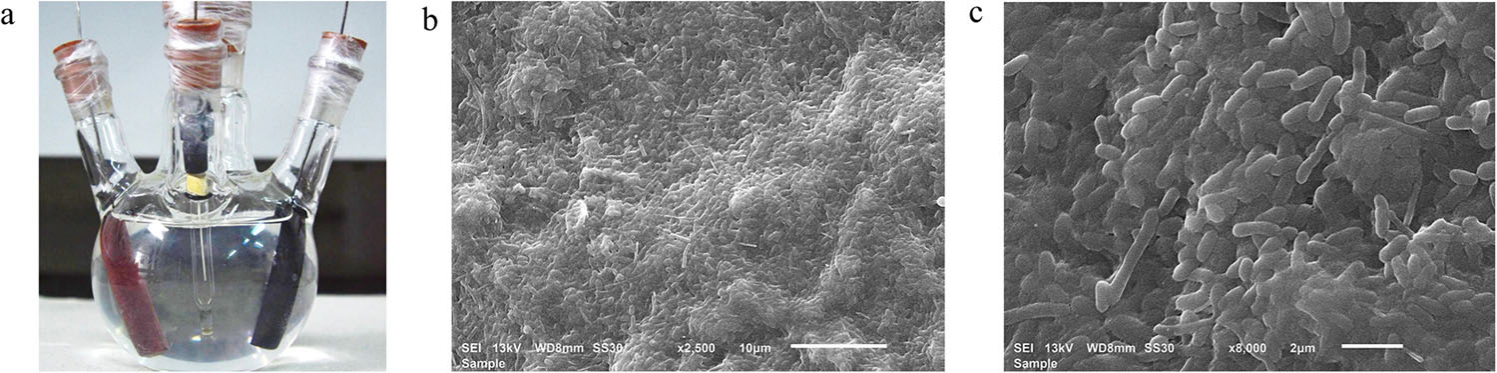
Images of the acetate-fed electroactive biofilms enriched at 0.2 V. Digital (**a**) and SEM images (**b**, **c**).

The digital images showed the appearance of brownish colored growth or biofilm at the electrode surfaces in both acetate- and lactate-fed reactors (Fig. 3a and Supplementary Fig. 6). The biofilm color appears quite similar to the known EAMs, such as *Geobacter* spp.^41^, *Geoalkalibacter* spp.^46^, and also the *Geobacter* sp.-dominated enriched mixed-culture biofilms^47^, growing at the electrodes. The scanning electron microscopy (SEM) imaging of these electrodes revealed the presence of mostly typical rod-shaped microbial cells and uniform biofilm coverage over the electrode surfaces (Fig. 3b, c and Supplementary Fig. 6). These observations, along with the CA results, confirm the growth of microbial EABs at the electrode surfaces under a haloalkaline environment.

The protein content measurements (Supplementary Methods) revealed that most microbial biomass was present at the electrode surface (up to 3.5 and 2.56 mg/L for acetate- and lactate-fed EABs, respectively) in comparison to the bulk phase or suspension (up to 0.8 mg/L in both acetate- and lactate-fed reactors).

### 16S-rRNA amplicon sequencing-based analysis of the enriched haloalkaliphilic microbial EABs

**Fig. 4:**
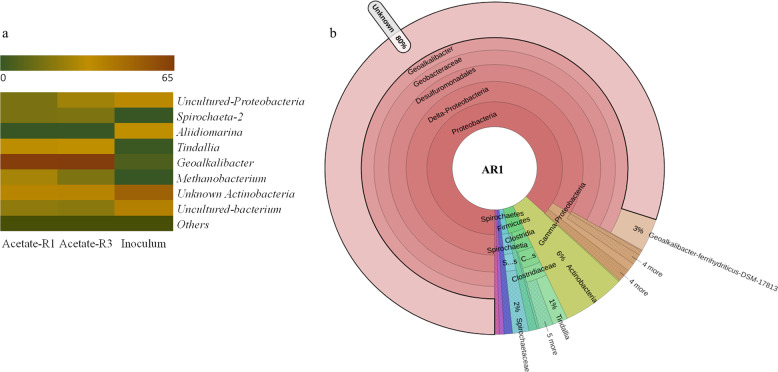
Microbial communities in the electroactive biofilms enriched at 0.2 V with acetate. **a** Heatmap showing the relative abundance of microorganisms at the genus level in the inoculum source and biofilms from two reactors (AR1 and AR3), and **b** Krona chart showing the taxonomy classification and relative abundances of microorganisms in the electroactive biofilm from reactor AR1.

**Fig. 5:**
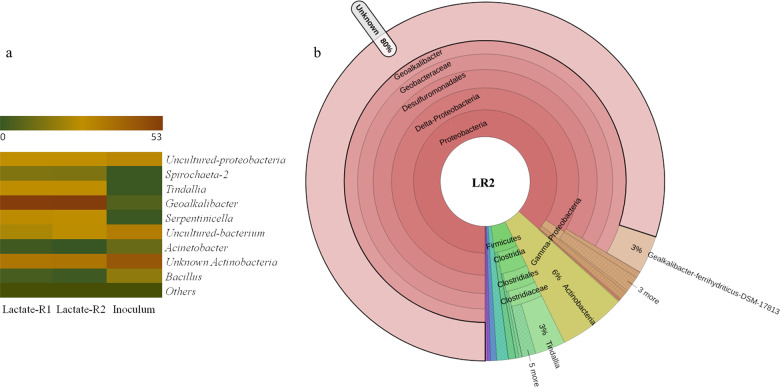
Microbial communities in the electroactive biofilms enriched at 0.2 V with lactate. **a** Heatmap showing the relative abundance of microorganisms at the genus level in the inoculum source and biofilms from two reactors (LR1 and LR2), and **b** Krona chart showing the taxonomy classification and relative abundances of microorganisms in the electroactive biofilm from reactor LR1.

The 16S-rRNA amplicon sequencing-based analysis revealed the relative abundance of mostly similar microbial communities, but at slightly different abundance levels in EABs enriched at 0.2 V with acetate and lactate substrates (Figs. 4 and 5, Supplementary Figs. 7 and 8, and Supplementary Table 1). The relative abundance data are discussed based on the average operational taxonomic units (OTUs) obtained with the EAB samples from two replicate reactors. The most dominant microbial communities in the sediment inoculum, namely, unknown *Actinobacteria*, uncultured-bacterium, uncultured-proteobacteria, *Aliidiomarina*, and *Bacillus*, were present at a relative abundance of 36.9%, 13.94%, 7.73%, 3.18%, and 1.65%, respectively. However, except for *Actinobacteria*, the relative abundance of other microbial groups decreased to <3 % in the enriched EABs with both acetate and lactate substrates. It suggests the inability of most of these microorganisms to grow by using the electrode as the terminal electron acceptor under anaerobic conditions. Whereas the microorganisms that got enriched in EABs were present at very low relative abundances in the original sediment inoculum source (as discussed further below). For instance, *Geoalkalibacter*, the most dominant genus in the enriched EABs, was present at only 0.61% relative abundance in the inoculum source.

### Microbial community composition in EABs enriched with acetate

*Deltaproteobacteria* was the most dominant microbial class with 65.62 ± 0.11% relative abundance followed up by *Clostridia* (12.4 ± 0.5 %), *Gamma-proteobacteria* (5.04 ± 0.2 %), *Spirochetes* (3.1 ± 0.12 %), and *Alpha-proteobacteria* (1.6 ± 0.11 %) in the case of acetate-grown EABs. At the order level, *Desulfuromonadales* was the dominant group at 64.8 ± 0.11% relative abundance, followed by *Clostridiales* at 10.94 ± 0.9% and *Spirochaetaes* 3.5 ± 0.7% abundances. Among the classes mentioned above, *Geobacteraceae* was the most dominant family (64.15 ± 0.01%) followed by *Clostridaceae* (7.2 ± 0.5%), *Spirochaetaceae* (3.0 ± 0.01%), unknown (3.0 ± 0.2%), ML635J-40-aquatic group (1.4 ± 0.6%), and *Izimaplasmataceae* (1.1 ± 0.14%). Further analysis at the genus level revealed 63.82 ± 0.5% of *Geoalkalibacter*, followed by an unknown *Actinobacteria* genus at ~10.5 ± 0.4% and *Tindalia* at 2.64 ± 0.2% relative abundances (Fig. 4 and Supplementary Table 1). Other OTUs belonged to uncultured-*Proteobacteria* and *Spirochaeta* genera at 3.26 ± 0.5% and 1.35 ± 0.05% relative abundances, respectively. *Methanobacterium* and *Pseudomonas* were also present, but at low relative abundance levels of 1.7 ± 0.4% and 1.08 ± 0.5%, respectively. At the species level, >80% of OTUs belonged to unknown species of the *Geoalkalibacter* genus. A very low relative abundance of known EAMs, such as *G. ferrihydriticus* (3.8 ± 0.12%) and *G. subterraneus* (0.012 ± 0.02%) was present in the enriched EABs. Uncultured spp. belonging to *Actinobacteria* genera were also present at 6.1 ± 0.18% relative abundance in this case.

### Microbial community composition in EABs enriched with lactate

In the case of lactate, barring a few exceptions, a similar pattern as that of acetate-grown EABs, in terms of community composition starting from the class to the species level, was observed, as elaborated further below. *Deltaproteobacteria* was the most dominant microbial class at 54.65 ± 0.14% relative abundance, followed up by *Clostridia* (25.8 ± 1.3%), *Gamma-Proteobacteria* (3.54 ± 0.2%), *Alpha-Proteobacteria* (1.3 ± 0.14%), and *Spirochetes* (3.3 ± 0.1%). At the order level, *Desulfuromonadales* (53.32 ± 0.6%), *Clostridiales* (24.94 ± 1.2%), and *Spirochaetes* (3.4 ± 0.13%) were the dominant groups. At the family level, *Geobacteraceae* (52.7 ± 0.3%), *Clostridaceae* (16.9 ± 1.2%), *Spirochaetaceae* (3.34 ± 0.1%), unknown (7.4 ± 0.6%), ML635J-40-aquatic group (1.8 ± 0.04 %), and *Izimaplasmataceae* (1.4 ± 0.4%) were the dominant groups. In this case, also *Geoalkalibacter* was the most dominant genera at 52.7 ± 0.3% relative abundance followed by the unknown *Actinobacteria* genus (18.12 ± 0.5%), *Serpentinicella* (5.0 ± 1.12%), *Tindalia* (4.0 ± 0.02%), uncultured-*Proteobacteria* genus (5.07 ± 0.04%), and *Spirochaeta* (1.4 ± 0.007%). *Serpentinicella* genus was present only in the lactate-grown EABs. Interestingly, ~80% unknown *Geoalkalibacter* spp. also got enriched in lactate-grown EABs (Fig. 5 and Supplementary Table 1), and the known electroactive *G. ferrihydriticus* (2.91 ± 0.08%) and *G. subterraneus* (0.012 ± 0.02%) were present in very low relative abundances, like that of acetate-grown EABs. About 8.5 ± 0.34% OTUs belonged to uncultured species of *Actinobacteria* genera.

Overall, the community data suggest that the electrochemical cultivation approach, along with the specific substrate conditions, led to the selection and enrichment of a few dominant microorganisms capable of respiring and growing, using an electrode as the electron acceptor under haloalkaline conditions. Also, the enriched EABs with acetate and lactate substrates showed similar species richness and unique identified species, as shown in chao1 and observed species count graph, respectively (Supplementary Figs. 9 and 10). It is well in agreement with the species abundance data, as discussed above.

## Discussion

Supplementary Table 2 summarizes the comparative overview of the reported EAMs under normal, saline, alkaline, and haloalkaline conditions. In this study, 548 ± 23 µA/cm^2^ current density was achieved in the case of acetate-fed electroactive haloalkaliphiles grown at 23 °C. It is much higher than the reported electroactive microbes enriched using the inoculum sources from the extreme haloalkaline environments, and close to a few known exoelectrogens tested under a closely related set of experimental conditions. For instance, enriched microorganisms from the haloalkaline Texcoco lake inoculum source achieved 128.1 µA/cm^2^ at an applied electrode potential of −0.111 V with pH 9.0, 13.5 g NaCl/L, and acetate substrate conditions^30^. A high current density of 4740 µA/cm^2^ has been reported with the adapted culture dominated by *Pseudomonas and Desulfuromonas* spp. at pH 10 and 12.6 g NaCl/L conditions, and applied electrode potential of −0.205 V (ref. ^29^). It should be noted here that this culture was not enriched from the native haloalkaline environmental source; it was rather adapted to such conditions. A few pure culture isolates of haloalkaliphilic strains have been tested for their electroactivity at haloalkaline or saline or alkaline conditions. These include, for instance, *N. magadii* and *Geoalkalibacter* spp*. N. magadii*, which is a high salt-tolerant strain, has been reported to produce a very low current density of 22 µA/cm^2^ at pH 10 and 200 g/L salinity conditions^28^. *Geoalkalibacter subterraneus* has been reported to produce 760 µA/cm^2^ at 0.239 V applied potential with 35 g NaCl/L and 7 pH conditions^17^. At pH 7 and 17 g/L salinity conditions, up to 330 and 506 µA/cm^2^ at the electrodes polarized at 0.239 and −0.2 V, respectively, have been reported with *G. subterraneus*^21,27^. Another *Geoalkalibacter* strain named *G. ferrihydriticus* has been reported to produce 830 µA/cm^2^ current density at pH 9, but with low salinity of 1 g/L at an applied potential of −0.2 V (ref. ^21^). A highly salt-tolerant microorganism *Haloferax volcanii* has been reported to produce 50 µA/cm^2^ current density at a high salinity of 144 g/L, but at a neutral pH condition^28^.

It should be noted here that most of the representative studies mentioned in Supplementary Table 2 were conducted at much higher incubation temperatures of 30 °C and above. As has been reported earlier, the incubation temperature in the mesophilic range can impact the bioelectrocatalytic performance of the EAMs^48,49^. Both the microbial growth and catalytic activity increases exponentially up to, and decreases beyond the optimum temperature^50^. Hence, for a direct comparison of the bioelectrocatalytic performance with the reported studies, a representative CA experiment was conducted with the acetate-fed enriched haloalkaliphilic electroactive microbial culture at 30 °C (Supplementary Fig. 4). It delivered up to 579 µA/cm^2^ current density, which is close to not only the tested, extreme haloalkaliphilic electroactive microbial cultures, but also known model electroactive microbes studied at the routinely used neutral pH and low salinity conditions (Supplementary Table 2). These observations suggest that the enriched electroactive haloalkaliphiles in this study are efficient for bioelectrocatalytic current generation or electroactivity compared to the known EAMs.

The electrochemical characterization revealed the direct mode of electron transfer from microbes to the electrode and presence of two prominent redox moieties, with the mean formal potentials of 0.183 and 0.333 V in the enriched haloalkaliphilic microbial EABs. The putative redox-active moieties are most likely the outer membrane-bound molecules or proteins of the EAMs^51^. The known EAMs including *Geobacter* spp., as well as the mixed-culture EABs dominated by *Geobacter* sp. show similar CV behavior and at least two prominent redox-active moieties^43,45,52^. What is interesting in the case of enriched electroactive haloalkaliphiles in this study is the redox-active moieties with higher (positive) formal potentials than the reported ones, so far for not only the halophilic or alkaliphilic exoelectrogens, but also the model exoelectrogens, such as *Geobacter* and *Shewanella* spp. (Table 2). In the closely related strains of *G. ferrihydriticus*, redox moieties active at 0.080 and −0.013 V (ref. ^21^), and for *G. subterraneus* at −0.402 and −0.383 V (ref. ^27^) have been reported. In the case of most well-studied exoelectrogen *G. sulfurreducens*, redox-active components with the formal potentials within a range of −0.350 to −0.420 V have been consistently reported^43,52–56^. For *Shewanella oneidensis* MR-1, which is another well-studied exoelectrogen, redox-active moieties with the formal potentials of −0.159, −0.305, −0.343, −0.380, and −0.405 V have been reported^57–59^. These are attributed mainly to the outer membrane cytochromes (OMCs)^57,58^. Putative OMCs or any other electron transport chain components with high reduction potentials, as observed in this study, have not been reported for the EAMs and also in any other microbial systems^51^, to the best of our knowledge. If the Nerstanian behavior of redox molecules is taken into account, and the formal potentials of the redox-active components observed in this study are recalculated at pH 7, the values are even higher (i.e., 0.334 and 0.480 V at pH 7). It thus implies that the enriched haloalkaliphilic EAMs in this study most likely possess some unreported membrane components that are involved in EET and, in turn, their respiratory processes under extreme growth conditions. Further work is warranted on isolating the most dominant microorganisms from the enriched EABs followed by investigating their EET mechanisms and associated components.

**Table 2:** An overview of the midpoint or formal potentials of the putative redox-active components or moieties, or outer membrane proteins/cytochromes involved in the electron transfer process to the electrode in different exoelectrogenic microorganisms.

S. no.	Microorganisms	Formal or midpoint potential of the redox-active moieties		References
		V vs. Ag/AgCl	V vs. SHE	
1.	Model exoelectrogens tested at neutral pH and normal salinity conditions			
	a. *Geobacter sulfurreducens*	−0.412	−0.207	^50^
	b. *Geobacter sulfurreducens*	−0.376	−0.171	^51^
	c. *Geobacter sulfurreducens*	−0.350	−0.145	^49^
	d. Mixed biofilm dominated with *Geobacter sulfurreducens*	−0.200	0.005	^52^
	e. *Shewanella oneidensis* MR-1	−0.445	−0.24	^55^
	f. *Shewanella oneidensis* MR-1	−0.405	−0.2	^54^
	g. *Shewanella oneidensis* MR-1	−0.305	−0.1	^54^
	h. *Shewanella oneidensi*s MR-1	−0.343	−0.138	^54^
	i. *Shewanella oneidensis* MR-1	−0.380	−0.175	^54^
	j. *Shewanella oneidensis* MR-1	−0.159	0.046	^53^
	k. *Thermincola ferriacetica*	−0.332	−0.127	^19^
2.	Exoelectrogens tested at neutral pH and high salinity			
	a. *Geoalkalibacter subterraneus*	−0.19	0.015	^17^
	b. *Geoalkalibacter subterraneus*	−0.401, −0.382	−0.196	^23^
	c. *Haloferax volcanii*	−0.300, 0.100	−0.095, 0.305	^24^
3.	Exoelectrogens tested at high saline–alkaline conditions			
	a. *Geoalkalibacter ferrihydriticus*	−0.21	−0.005	^21^
	b. Mixed-culture biofilm	−0.176, −0.131	0.029, 0.074	^26^
	c. *Geoalkalibacter* spp.-dominated biofilm	0.18, 0.33	0.385, 0.535	This study

*SHE* standard hydrogen electrode.

The 16S-rRNA amplicon sequencing of the enriched EABs revealed >80% dominance by unknown *Geoalkalibacter* spp. in the case of both acetate and lactate substrates. *Geoalkalibacter* has evolved as a unique phylogenetic branch within the *Geobacteraceae* family. Most of the genera within the *Geobacteraceae* family exhibit the capabilities to respire on insoluble, extracellular terminal electron acceptors, namely Fe (III) and Mn (IV)^60^. The prominent genus *Geobacter* is found mostly in freshwater sediment environments, while *Desulfuromusa* and *Desulfuromonas* genera inhabit mostly halophilic environments^60^. *Geoalkalibacter* spp. has an additional advantage of tolerance to alkalinity besides being halotolerant. The *Geoalkalibacter* genus thus seems to be more related to *Desulfuromusa* and *Desulfuromonas* than the *Geobacter* genus^61^. It has a high sequence similarity (~90%) to halophilic *Desulfuromonas*^61^. So far, only two pure culture isolates belonging to this family have been reported to be electroactive. These include *G. ferrihydriticus* and *G. subterraneus*^17,21,27^. Both these species were present in the enriched EABs, but at a low abundance of <4% in this study. The presence of >80% of the unknown *Geoalkalibacter* spp. thus suggests enrichment of the unreported electroactive haloalkaliphiles.

Those microorganisms which have been reported to be electroactive at either alkaline or saline or both conditions, such as *Desulfuromonas soudanesis*^62^, *Shewanella marisflavi* EP1 (ref. ^63^), *Halanaerobium praevales*^64^, and *N. magadii*^28^ were not detected in the enriched microbial EABs in this study. In addition to the *Geoalkalibacter* genus, other dominant microbes that got enriched with acetate belong to unknown *Actinobacteria* genera (10.5 ± 0.4%), followed by *Tindallia* (2.64 ± 0.2%), *Methanobacterium* (1.7 ± 0.4%), and *Spirochaeta-2* spp. (1.4 ± 0.05%). In the case of lactate, in addition to unknown *Actinobacteria genera* (18.12 ± 0.5%) and *Tindallia* (4.0 ± 0.02%), *Serpentinicella* was the dominant genera at 1.4 ± 0.007% abundance. None of these genera have been reported to be electroactive so far. Among these, *Tindallia*, a fermentative alkaliphilic anaerobe, is known to reduce iron^65^. It can thus be proposed to possess electroactivity, which, however, needs to be confirmed through further electrochemical tests with its pure strains. *Serpentinicella* is an alkaliphilic anaerobe, which belongs to the *Clostridaceae* family. *Serpentinicella* has been reported to be an obligate user of lactate, crotonate, and pyruvate as carbon and energy sources instead of acetate and other sugars^66^. Its presence in the lactate-fed EABs thus clearly suggests its role in oxidizing lactate to produce acetate, and in turn, make it available for further oxidation by the electroactive *Geoalkalibacter* spp. It is an example of syntrophic interactions between different microbial communities in such mixed-culture-based EABs. *Geoalkalibacter* spp. has also been reported to oxidize lactate directly to reduce Fe (III) and Mn (IV)^61^. Based on this metabolic capability and its dominance in the lactate-fed EAB, it can be hypothesized that *Geoalkalibacter* spp. are most likely able to oxidize lactate completely (without forming any intermediates) and reduce electrode. However, to confirm this metabolic capability, further work needs to be conducted with the pure culture *Geoalkalibacter* isolates from the enriched EAB.

Overall, >15% dominance by the unknown genera besides the presence of >80% unknown *Geoalkalibacter* spp. in both the acetate- and lactate-grown EABs suggest enrichment of the unreported exoelectrogenic microorganisms in this study. It warrants further follow up work on isolation, characterization, and identification of these enriched haloalkaliphilic EAMs.

In this study, the electrochemical cultivation approach resulted in the successful enrichment of microbial EABs at all but −0.2 V applied potentials under the haloalkaline conditions. Best-performing haloalkaliphilic EABs in terms of maximum bioelectrocatalytic current densities were obtained at 0.2 V applied potential with both acetate and lactate substrates. SEM imaging confirmed the microbial growth and biofilm formation over the electrode surface. The enriched EABs possess redox-active moieties with high, positive formal potentials that have not been reported for any known EAMs so far. Also, the EABs were found to be dominated by unknown *Geoalkalibacter* spp. Thus, this study broadens the diversity of the known EAMs and the formal potentials of the components putatively involved in EET. It also provides a crucial platform to investigate the diversity of EAMs from different other extreme environments, which has barely been explored thus far. By reporting on haloalkaliphilic microbial EABs, it contributes to the advancement of the extreme electromicrobiology field, and opens up opportunities for both basic and applied research. For instance, further work on isolation and characterization of the dominant haloalkaliphilic exoelectrogens in the enriched EABs might lead to the expansion of the database of the microorganisms possessing extreme electroactivity and detailed investigations on the EET mechanisms and components. For applied research, the prominent niche-specific applications that the haloalkaliphilic EAMs can offer include (i) harnessing energy, in the form of either electricity or hydrogen, from haloalkaline environments, urine and wastewaters from aquaculture, meat-processing, tannery, and petro-refinery caustic industries using microbial fuel or electrolysis cells^21,29,67^, and (ii) the production of value-added chemicals from CO_2_ via microbial electrosynthesis process^68^.

## Methods

All microbiological experiments were conducted under anaerobic conditions at an incubation temperature of 23 ± 2 °C. If not stated otherwise, all electrode potential data are referred to vs. Ag/AgCl (3.5 M KCl) reference electrode (0.205 V vs. SHE (standard hydrogen electrode)). All numerical data are presented as averages along with uncertainties based on at least three replicate experiments or sample analysis, if not mentioned otherwise.

### Sediment sampling and characterization

We collected the sediment samples from the Lonar lake (19° 58′43.81″ N and 76° 30′29.31″ E, Buldhana district, India) during winter and monsoon (i.e., January and August 2019) seasons. Sediment sampling and characterization details are presented in Supplementary Methods.

### Reactor setup, microbial growth medium, and inoculum source

For electrochemical cultivation or enrichment experiments, potentiostatically controlled three-electrode configuration reactors (250 mL capacity) were used^69^. Each reactor hosted two graphite rods (projected surface area of 16.485 cm^2^) as the working and counter electrodes and an Ag/AgCl (3.5 M KCl, 0.205 V vs. SHE) reference electrode. Before use, the graphite electrodes were pretreated using the acid–alkali method^70^, and heating at 400 °C in a muffle furnace (Nabertherm, Germany) for 5 min followed by polishing with grit sandpaper (P180). Titanium wire (99.999% pure metal basis, 1 mm thick, Alfa Aesar, USA) was used as the current collector and to establish connection between the electrodes and potentiostat channel terminals.

A 200 mL of modified M9 medium with pH 9.5 and salinity 20 g/L served as the growth medium or electrolyte in the electrochemical reactors. It contained the following (per L of distilled water): 4.33 g Na_2_HPO_4_, 2.69 g NaH_2_PO_4,_ 20 g NaCl, 4.3 g Na_2_CO_3_, 0.13 g KCl, 0.31 g NH_4_Cl, 12.5 mL vitamins, 12.5 mL trace elements, and 10 mM acetate or lactate, as the sole carbon and electron donor source. It lacked any known soluble or insoluble electron acceptor. Before use, the medium was made anaerobic by sparging with 99.999% inert N_2_ gas (Sigma Gases, India) at least for 20 min. The sediment samples obtained from the Lonar Lake served as the microbial inoculum source (Supplementary Methods). Before conducting experiments, the reactor headspace was sparged with N_2_ gas, and all openings were sealed with gastight butyl rubber stoppers for maintaining anaerobic conditions.

### Electrochemical enrichment of the electroactive microorganisms

All enrichment experiments were conducted in at least triplicates under potentiostatically controlled conditions (VMP3 multichannel electrochemical workstation, BioLogic Science Instruments, France). The reactors with acetate and lactate are denoted as ARn and LRn, respectively. A and L represent acetate and lactate, respectively, R represents reactor, and *n* is the number of reactor replicate. The electrochemical experiments involved applying different potentials, viz. −0.2, 0.0, 0.2, and 0.4 V at the working electrode, and monitoring the substrate oxidation current at a fixed time interval of 2 min using CA technique. The purpose of polarizing electrodes at different set potentials was to use them as an analog or proxy to different natural electron acceptor conditions^12,38^. Other parameters such as pH and substrate concentration in the medium were monitored regularly. The CA experiments were conducted for at least three batch cycles by replenishing the spent medium with a complete fresh M9 medium. Two control experiments, namely, abiotic connected, i.e., electrochemically connected but uninoculated reactor and biotic unconnected, i.e., electrochemically unconnected but inoculated reactor, were also conducted to compare and confirm the (bio)electrocatalytic current generation. The electric current response data are presented by normalizing it with the projected surface area of the electrode. CE was calculated to know the amount of chemical energy converted into the electric current by the enriched microbial EABs (Supplementary Methods).

Further detailed characterization of the best-performing haloalkaliphilic microbial EABs in terms of bioelectrocatalytic substrate oxidation current production was conducted via electrochemical, microscopic, and 16S-rRNA amplicon sequencing techniques, as discussed in the following sections. Furthermore, for comparing the bioelectrocatalytic performance of the haloalkaliphilic EABs at commonly used incubation temperature in other studies, a representative CA experiment was conducted with the acetate-grown enriched culture at 30 °C.

### Characterization of the enriched haloalkaliphilic microbial EABs

The best-performing EABs, in terms of maximum bioelectrocatalytic current response, were chosen for further characterization based on electrochemical, SEM, and 16S-rRNA amplicon sequencing tools and techniques.

### Electrochemical characterization

The cyclic voltammetry technique was used to understand the type of electron transfer mechanism, and the formal potentials of the redox-active components involved in the electron transfer process by the enriched EABs^43^. CVs were recorded under different conditions in a potential window of −0.4 to +0.6 V at 1 mV/s scan rate. These include, before and after microbial inoculation (control CVs), during substrate turnover (i.e., at the condition of bioelectrocatalytic current response linked to substrate oxidation) and non-turnover conditions (in the absence of substrate, thus no catalytic current response) at the end of CA experiments, and in a filtered spent medium with the new electrodes.

### Scanning electron microscopy analysis

The bioelectrode samples (electrodes with the microbial biofilm) were fixed by incubating overnight in a fixative solution (2% glutaraldehyde and 2.5% paraformaldehyde) at 4 °C. Postfixation was done by incubating the samples in 1% osmium tetraoxide for 90 min, followed by dehydration. For this purpose, the samples were placed in different dilutions of ethanol (30, 50, 70, 80, 90, and 100%) sequentially (20 min in each dilution). Then the samples were dried overnight in a silica desiccator. Finally, the samples were coated or sputtered with gold nanoparticles by JEOL JEC-1600 Auto-Fine Coater (JEOL Ltd., Japan) at 20 mA for 45 s, and analyzed by using JEOL JSM-6010PLUS/LS scanning electron microscope (JEOL Ltd., Japan).

### 16S-rRNA amplicon sequencing-based analysis of the enriched haloalkaliphilic microbial EABs

The genomic DNA was extracted using DNeasy® PowerSoil® Pro kit (Qiagen, Germany) from the inoculum source and the EABs grown at 0.2 V applied potential, with acetate and lactate substrates from two replicate reactors. The isolated DNA samples were quantified by absorbance measurement using Nanodrop (Genova Nano 4359, Jenway, Cole-Parmer, UK), Qubit fluorimeter (V.3.0, Thermo Fischer Scientific, USA), and the integrity of samples was analyzed by agarose gel electrophoresis. The isolated DNA samples were used as a template for synthesizing 16S rRNA sequences. The V3 and V4 regions of synthesized 16S rRNA sequences were amplified by using specific V3 forward primer 5′-CCTACGGGNBGCASCAG-3′ and V4 reverse primer 5′-GACTACNVGGGTATCTAATCC-3′, as described in the Illumina 16S-rRNA amplicon sequencing preparation guide (https:support.illumina.com/documents/documentation/chemistry_documentation/16s/16s-metagenomic-librabry-prep-guide-15044223-b.pdf). The amplified products were checked and analyzed on 2% agarose gel. It was followed by library generation using NEBNext Ultra DNA library preparation kit. Later, Agilent 2200 TapeStation was used to estimate and quantize the library products. The chosen library was then processed through Illumina HiSeq 4000 machine to generate the more elongated sequence of 2 × 250 base pairs at AgriGenome Labs Pvt. Ltd. Cochi, India.

Initially, the raw data sequences were processed through In-House PERL script to trim the forward and reverse primer sequences, followed by merging the sequences to build consensus V3 and V4 using a FLASH program (version 1.2.11). Merging was done with a minimum overlap of 10 bp to a maximum overlap of 240 bp with zero percent mismatches. The resulted consensus was trimmed for removing the chimeras using the UCHIME-V11 tool (de novo chimera removing method) in the VSEARCH program. After this, the trimmed consensuses were used to pickup the OTUs, using the Uclust program already available in QIIME software. The generated raw sequences were grouped as OTUs or phylotypes at 97% (*p* < 0.03) identity. OTUs having more than five reads were used for further processing, and analysis and the rest of the OTUs were discarded. Then the sequences with the highest abundances within a cluster were selected as the consensuses/representative sequence for that OTU. These consensuses were aligned against the SILVA core set of sequences using the PyNAST program in QIIME1. The taxonomic identification and classification were done by using the RDP classifier program for mapping each representative sequence against the SILVA OTU database. Finally, all the sequences of a particular OTU multiplied by natural logarithm were used to plot Shannon diversity indexes explaining rarefaction for alpha diversity within the samples. Similarly, the metric calculation was performed using QIIME software to plot Chao1 diversity and observed species metrics. The beta diversity was estimated using the principal component analysis. Only the OTUs with ≥1% abundances were used to plot heatmaps and Krona charts using Origin Pro 2020 and Krona tools, respectively. The OTU sequences used to create heatmaps and Krona charts have been deposited to NCBI Sequence Read Archive (SRA) with SRR11014377–81 accession numbers.

### Reporting summary

Further information on research design is available in the Nature Research Reporting Summary linked to this article.

## Supplementary information

Supplementary information

Reporting Summary

## Data Availability

All data generated or analyzed during this study are included in this article and its Supplementary information file. All raw sequencing data are available on the NCBI archive with the project accession number PRJNA604728.
