# Supplementary material for: Microbial electroactive biofilms dominated by Geoalkalibacter spp. from a highly saline–alkaline environment
Source: NPJ Biofilms Microbiomes. 2020 Oct 13;6:38. doi: 10.1038/s41522-020-00147-7 (PMC7555509; doi:10.1038/s41522-020-00147-7)
Supplement: Supplementary file 1 — Supplementary information [file 41522_2020_147_MOESM1_ESM.pdf]

Supplementary Information

Microbial electroactive biofilms dominated by *Geoalkalibacter* spp. from a highly saline-alkaline environment

Sukrampal and Sunil A. Patil*

Department of Earth and Environmental Sciences, Indian Institute of Science Education and Research Mohali (IISER Mohali), Knowledge City, Sector 81, SAS Nagar, 140306, Punjab, India

*Email for correspondence: sunil@iisermohali.ac.in

Supplementary Methods

Sediment sampling and characterization

At least three peripheral locations of the Lonar Lake were selected for sampling to minimize uncertainties in the physicochemical characteristics of the sediment samples and to get the representative microbial inoculum source from the lake system. Sediment samples from up to a depth of ~1 ft. from the surface were collected in air-tight amber-colored sampling bottles of 1 L capacity. The sediment samples were analyzed immediately for physical (pH, conductivity, and salinity) and chemical (ammonium ions, orthophosphate, nitrate, sulfate, and chemical oxygen demand) parameters according to the standard protocols[1]. They were stored at 4 ºC in sealed containers when not in use.

Inoculum preparation

Approximately 4 g of the sediment sample was mixed in 40 ml of anaerobic medium, followed by sonication and centrifugation (at 8000 rpm for 10 minutes) to loosen the microorganisms from

solid particles. The resulting suspension was then used as the microbial inoculum source for enrichment experiments.

Coulombic efficiency

Coulombic efficiency (CE) was calculated to find out the efficiency of enriched electroactive microorganisms or biofilm to convert chemical energy into electrical energy, according to the following formula.

$$\mathrm{CE} = \frac{\mathrm{M}\sum_{0}^{ti} It}{\mathrm{Fb}\mathrm{V}_{\mathrm{An}}\Delta[S]}$$

Where ‘M’ represents the molecular weight of the electron donor/substrate; ‘F’ the Faraday’s constant; ‘b’ the number of electrons exchanged per substrate molecule; $\Delta[S]$ the change in the substrate concentration, V_{An} the working volume, and It represents the current over the specific time.

Protein Estimation

The protein content of the biomass enriched over the electrode surface and present in the suspension was estimated using Bradford assay, using Bovine Serum Albumin (BSA) as a standard.

Supplementary Figures

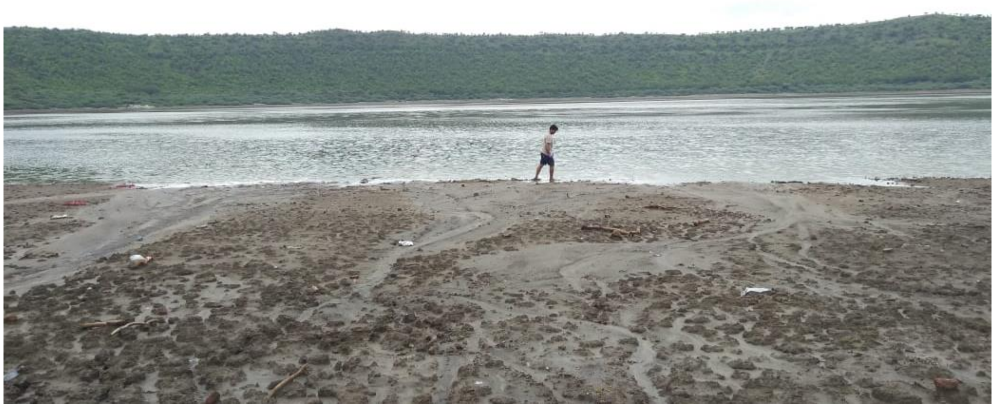

Supplementary Figure 1: Representative digital image of the Lonar Lake, the only saline-alkaline hypervelocity impact meteorite Crater Lake in basaltic rock in the world. (Geological Location: 19° 58'43.81" N and 76° 30'29.31" E; Buldhana District, India). Photo taken by the authors on the paper.

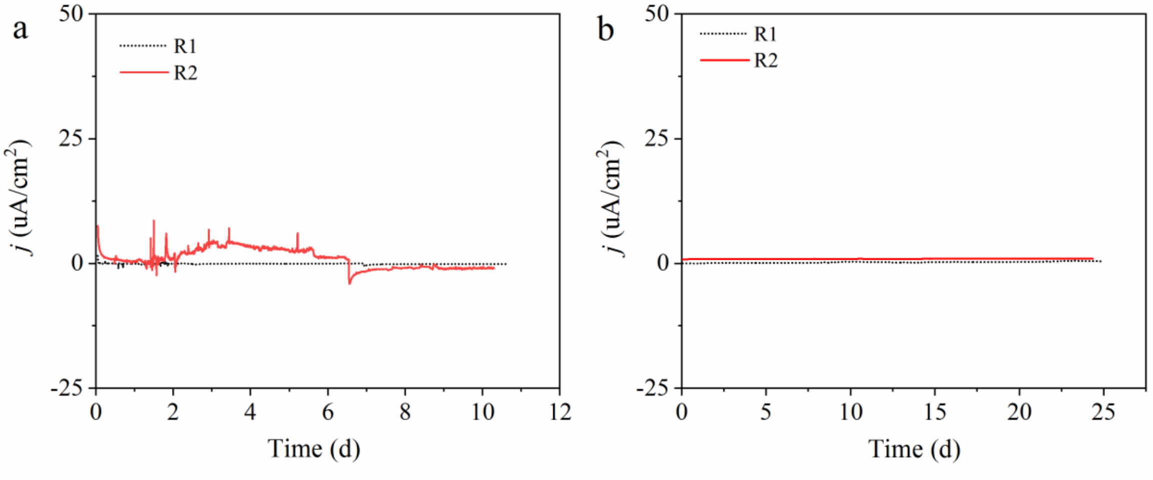

Supplementary Figure 2: Chronoamperometry (CA) profiles showing the bioelectrocatalytic current generation at the electrodes polarized at -0.2 V *vs.* Ag/AgCl (3.5 M KCl) in two replicate reactors fed with 10 mM (a) acetate and (b) lactate substrates.

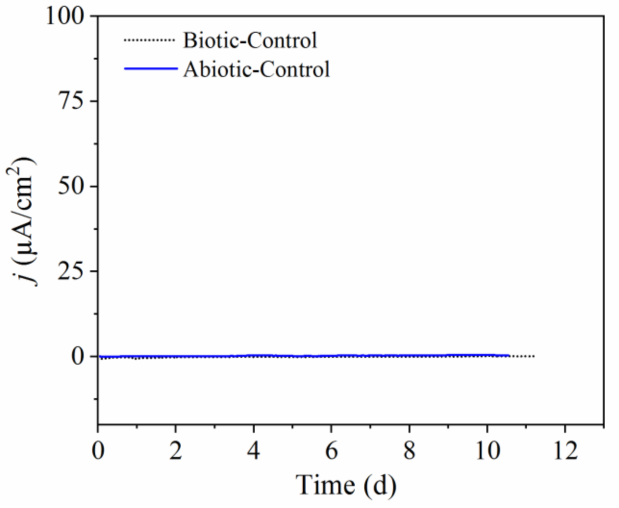

Supplementary Figure 3: Chronoamperometry (CA) profiles of the control experiments, namely, biotic-unconnected and abiotic connected (with the electrode polarized at 0.2 V *vs.* Ag/AgCl) with acetate (10 mM) substrate.

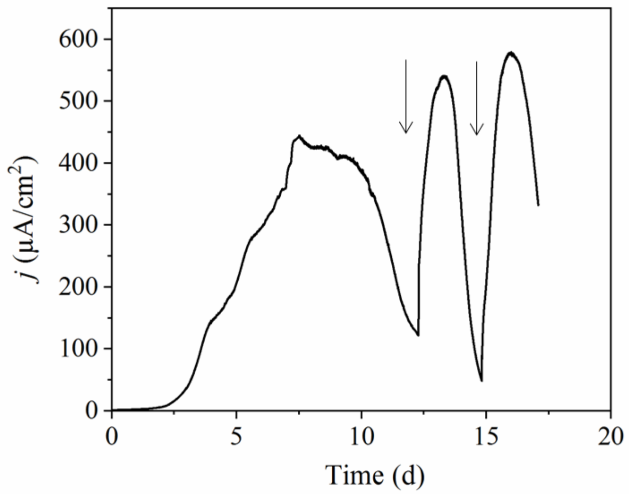

Supplementary Figure 4: Chronoamperometry (CA) profile of the acetate-grown enriched culture at 30 °C. (Electrode polarized at 0.2 V *vs.* Ag/AgCl). Arrows represent the medium replenishment.

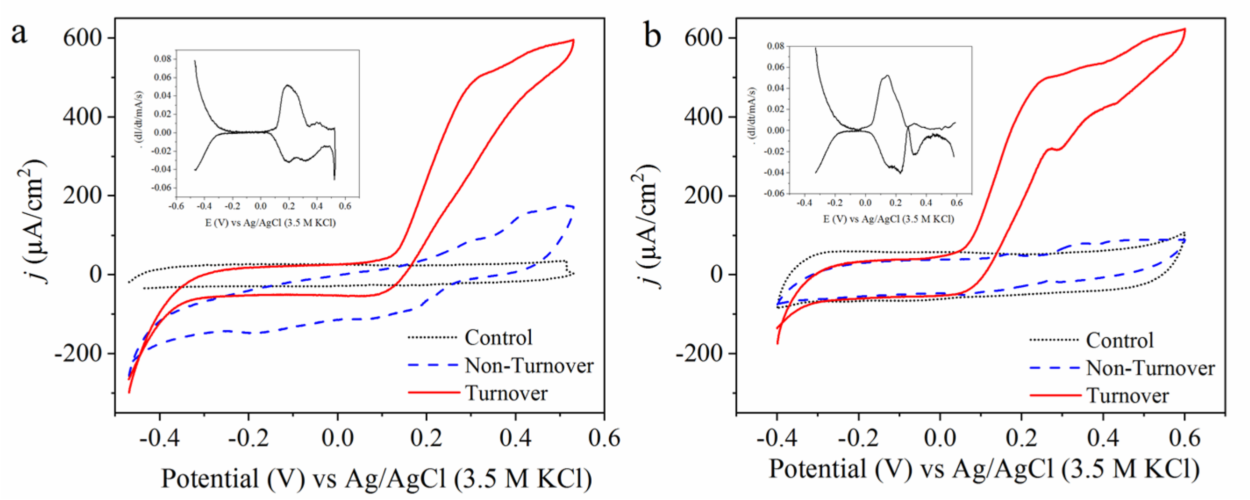

Supplementary Figure 5: Cyclic voltammograms obtained at different conditions at a scan rate of 1 mV/s for the (a) acetate-fed (AR3 replicate) and (b) lactate-fed biofilms (LR2 replicate).

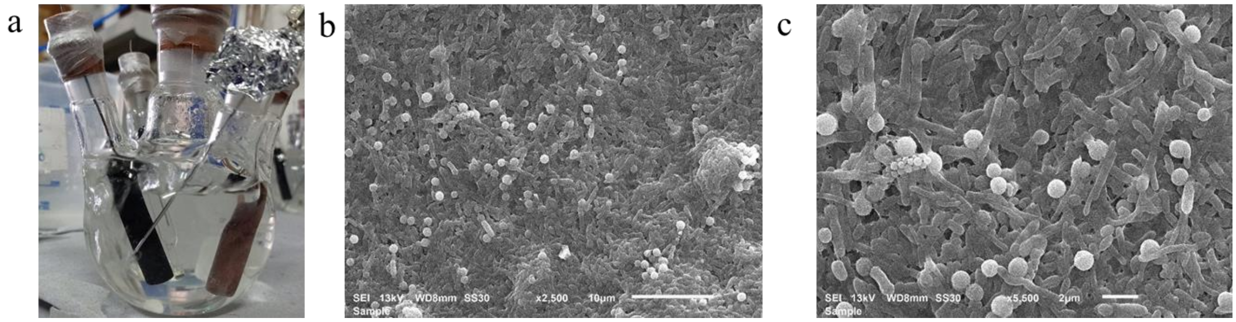

Supplementary Figure 6: Digital (a) and SEM images (b - scale bar 10 μm and c - scale bar 2 μm) showing the growth of electroactive microorganisms at the electrode surface polarized at 0.2 V *vs.* Ag/AgCl. Substrate: 10 mM lactate

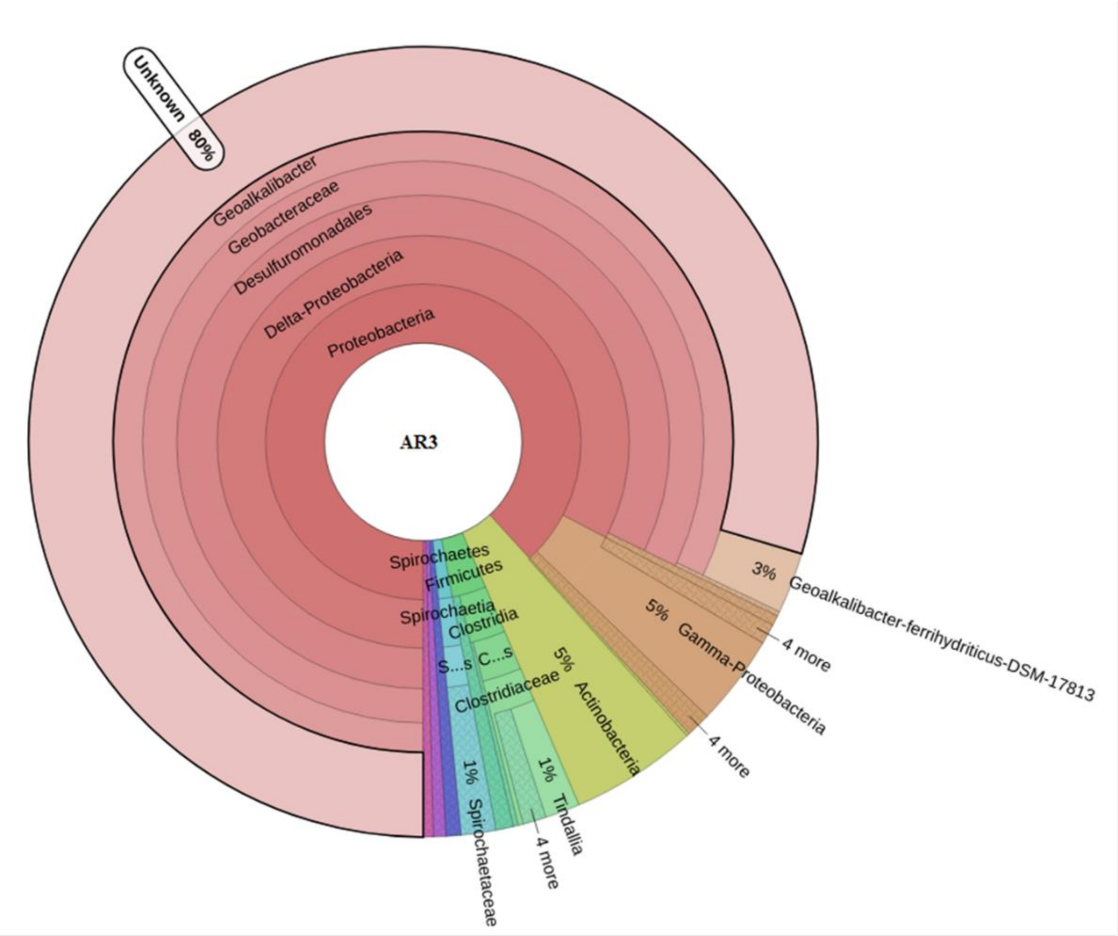

Supplementary Figure 7: Krona chart showing the taxonomy classification and relative abundances of the enriched haloalkaliphilic exoelectrogenic microorganisms at the electrode polarized at 0.2 V *vs*. Ag/AgCl in the acetate-fed replicate reactor AR_3.

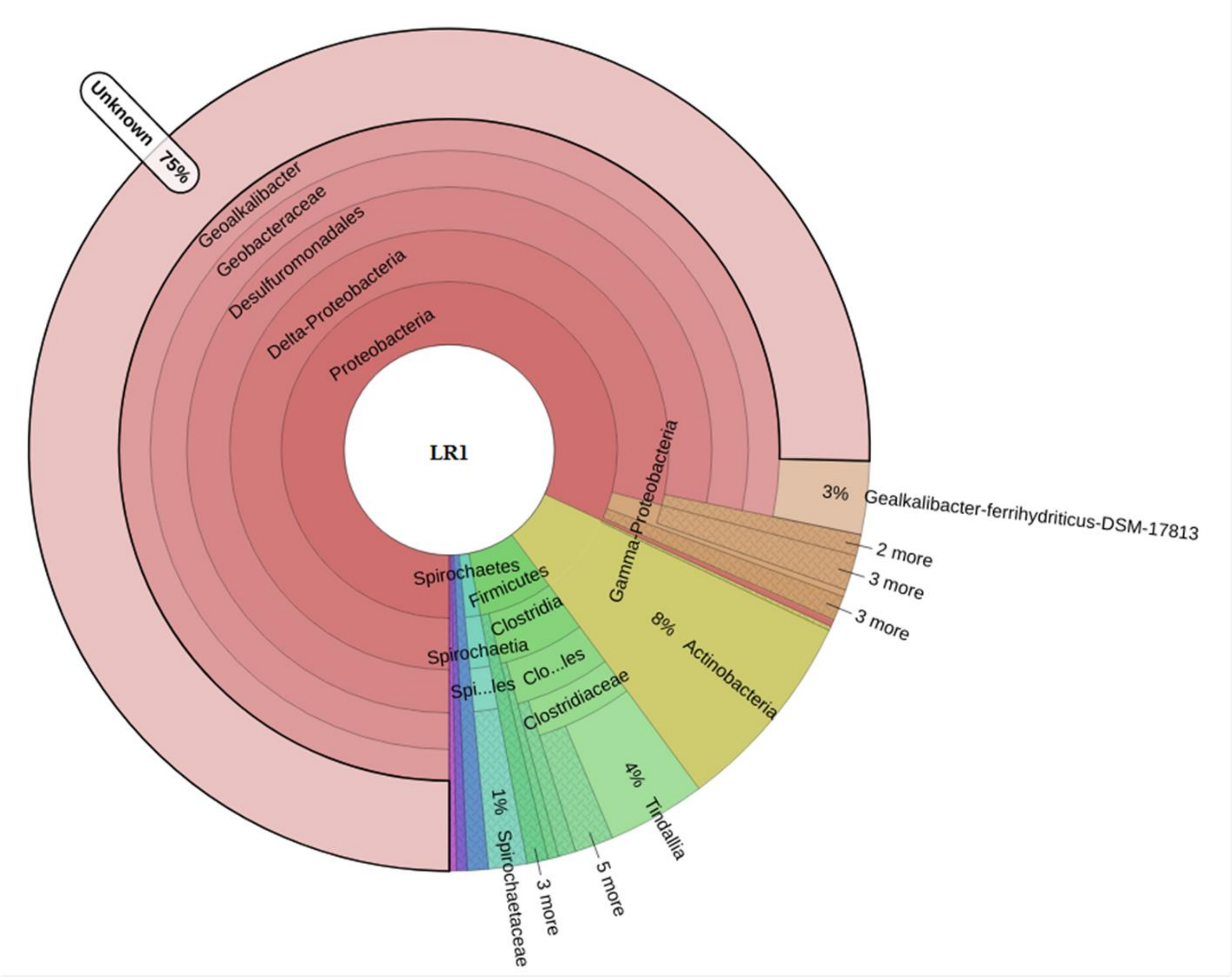

Supplementary Figure 8: Krona chart showing the taxonomy classification and relative abundances of the enriched haloalkaliphilic exoelectrogenic microorganisms at the electrode polarized at 0.2 V *vs*. Ag/AgCl in the lactate-fed replicate reactor LR_2.

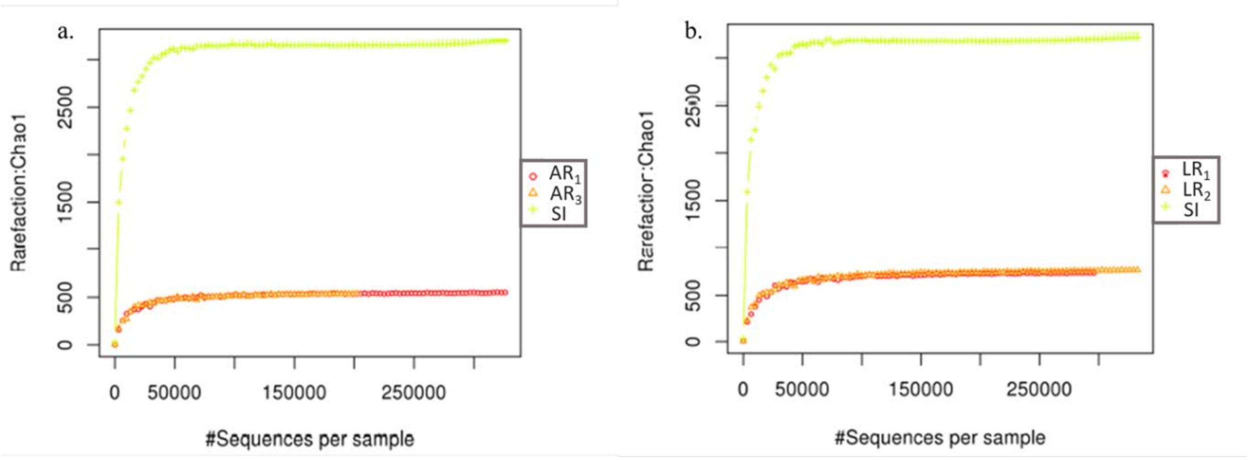

Supplementary Figure 9: Estimated species richness in the microbial electroactive biofilms enriched with a) acetate (AR_1 and AR_3 reactors) and **(**b) lactate (LR_1 and LR_3 reactors) substrates in comparison with species richness in the sediment inoculum (SI) source.

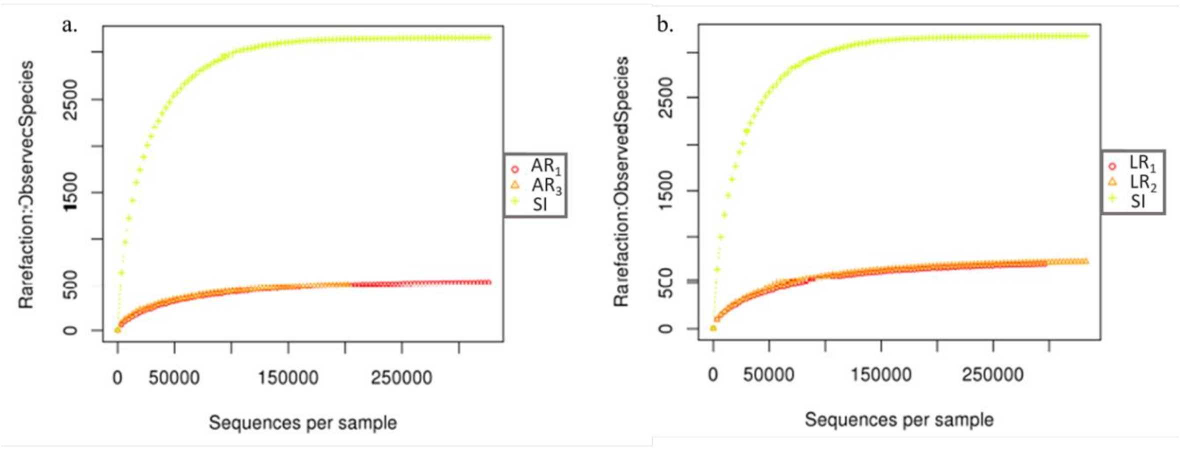

Supplementary Figure 10: The count of unique species identified in the microbial electroactive biofilms enriched with a) acetate (AR_1 and AR_3 reactors) and (b) lactate (LR_1 and LR_3 reactors) substrates in comparison with the sediment inoculum (SI) source.

Supplementary Tables

Supplementary Table 1: Relative OTUs abundance data of the enriched haloalkaliphilic microbial EABs with acetate (AR_1 and AR_3 reactors) and lactate (LR_1 and LR_2 reactors) substrates at different taxonomic levels.

S. No.	Taxonomic Levels	Relative Abundances (%)	
		Acetate-Fed EABs	Lactate-Fed EABs
1.	Phylum		
	a. Proteobacteria	72.2 ± 0.14	58.65 ± 1.7
	b. Firmicutes	13.07 ± 0.7	27.06 ± 1.33
	c. Actinobacteria	10.9 ± 0.14	10.7 ± 0.25
	d. Spirochaetes	3.1 ± 0.13	3.33 ± 0.1
	e. Others	≤ 1.00	≤ 1.00
2.	Class		
	a. Delta-Proteobacteria	65.62 ± 0.11	54.65 ± 0.14
	b. Gamma-Proteobacteria	5.04 ± 0.2	3.54 ± 0.2
	c. Bacteroidia	4.26 ± 0.11	3.78 ± 0.007
	d. Spirochaetia	3.105 ± 0.12	3.33 ± 0.1
	e. Alpha-Proteobacteria	1.6 ± 0.14	1.3 ± 0.14
	f. Clostridia	-	25.8 ± 1.3
	g. Others	≤ 1.00	≤ 1.00
3.	Order		
	a. Desulfuromonadales	64.82 ± 0.11	53.32 ± 0.6
	b. Clostriadales	10.94 ± 0.9	24.94 ± 1.21
	c. Spirochaetaes	3.5 ± 0.7	3.4 ± 0.13
	d. Bacteroidales	4.0 ± 0.014	-
	e. Others	≤ 1.00	≤ 1.00
4.	Family		
	a. Geobactereaceae	64.145 ± 0.01	53.32 ± 0.311
	b. Clostridiaceae	7.2 ± 0.5	16.89 ± 1.19
	c. Spirochaetaceae	3.0 ± 0.01	3.43 ± 0.10
	d. ML635J-40-aquatic group	1.4 ± 0.6	1.80 ± 0.05
	e. Unknown Family	3.0 ± 0.2	7.4 ± 0.6

	f. Izimaplasmataceae	1.1 ± 0.14	1.4 ± 0.4
	g. Others	≤ 1.00	≤ 1.00
5.	Genus		
	a. Geoalkalibacter	63.82 ± 0.5	52.7 ± 0.31
	b. Unknown Actinobacteria	10.47 ± 0.4	18.115 ± 0.54
	c. Tindallia	2.64 ± 0.2	4.0 ± 0.02
	d. Uncultured Proteobacteria	3.26 ± 0.48	5.07 ± 0.04
	e. Serpentinicella	-	5.0 ± 1.12
	f. Spirochaeta	1.35 ± 0.05	1.4 ± 0.01
	g. Methanobacterium	1.7 ± 0.43	-
	h. Others	≤ 1.00	≤ 1.00
6.	Species		
	a. Unknown	80.5 ± 0.47	80.13 ± 0.09
	b. Uncultured Actinobacteria	6.1 ± 0.18	8.46 ± 0.33
	c. Geoalkalibacter	3.87 ± 0.13	2.91 ± 0.08
	ferrihydriticus	0.012 ± 0.02	0.012 ± 0.02
	d. Geoalkalibacter subterraneus		
	e. Others[1]	≤ 1.00	≤ 1.00

[1]Note: Others are dominated by *Desulfobolus alkaliphilus, Pseudomonas salegens, Spirochaeta asiatica, Geobacter soli,* and *Clostridium aceticum* in the case of acetate-grown EABs, whereas by *Halomonas campisalis, Pseudomonas aeruginosa, Serpentinicella alkaliphila, Spirochaeta africana,* and *Geobacter soli* in the lactate-grown EABs.

Supplementary Table 2: A comparative overview of the bioelectrocatalytic current generation data by the model exoelectrogens and extreme electroactive microorganisms grown at neutral pH or high pH or high salinity or highly saline-alkaline conditions.

S. No.	Microorganism	Source	pH	Salinity (g/L NaCl)	Substrate or Electron donor	Applied E (V) vs. Ag/AgCl	Max. j ($\mu A/cm^2$)	Ref.
1.	Model exoelectrogens							
	a. Geobacter sulfurreducens	Previously operated MEC	6.8	-	Acetate	0.035	500	2
	b. Geobacter sulfurreducens	ATCC	6.8	-	Acetate	-0.005	340	3
	c. Geobacter sulfurreducens	ATCC	7	-	Acetate	0.300	315	4
	d. Geobacter sulfurreducens	DSMZ	6.8	-	Acetate	0.0	100	5
	e. Thermincola ferriacetica	DSMZ	6.95	1	Yeast Extract	-0.265	800	6
	f. Geobacter SD-1	Domestic Wastewater	7	-	Acetate	0.7	290 ± 29	7
	g. Shewanella oneidensis MR-1	Previously Modified Strain	7	5.8	Lactate	0.2	7.9	8
	h. Shewanella oneidensis MR-1	Previously Modified Strain	7	10	Lactate	MFC mode	74	9
	i. Shewanella oneidensis MR-1	ATCC	7	10	Lactate	MFC mode	176	10
2.	Exoelectrogens at neutral pH and high salinity							
	a. Geoalkalibacter subterraneus	DSMZ	7	35, 17	Acetate	0.245	506	11

b. Mixed-culture biofilm	Different Sediments[1]	7	20	Acetate	-0.30	892	12
c. Haloferax volcanii	ATCC	7	144	Yeast Extract	MFC mode	600	13
3. Exoelectrogens at high pH							
a. Mixed Biofilm dominated by Enterococcaceae and Geoalkalibacter spp.	Sludge	9.3	1	Acetate	0.8	0.25	14
4. Exoelectrogens at high saline-alkaline conditions							
a. Geoalkalibacter ferrihydriticus	Khadyn Lake, Russia	9.3	1	Acetate	-0.2	830	15
b. Mixed Culture Biofilm	Texcoco Lake, Mexico	9	13.5	Acetate	-0.105	128.1	16
c. Mixed-culture biofilm dominated by Pseudomonas and Desulfuromonas spp.	Previously operated MFC	10	12.6	Acetate	-0.205	4740	17
d. Natrialba magadii	Magadi Lake, Kenya	10	200	Citrate	MFC	22	13
e. Unknown Geoalkalibacter spp. dominated biofilm	Lonar Lake, India	9.5	20	Acetate	0.2	548±23	This Study
f. Unknown Geoalkalibacter spp. dominated biofilm	Lonar Lake, India	9.5	20	Lactate	0.2	437±17	This Study

Note - Except ref. 25 (23 ± 1ºC), 77 (20 ºC) and this study (23 ± 2 ºC), rest of the studies were conducted at an incubation temperature of ≥ 30 ºC.

MFC: Microbial fuel cell; MEC: Microbial electrolysis cell

[1]Superior, AZ, USA; Saline microbial mat from Cabo Rojo, PR, USA; saltwater sediment (mangrove swamp), Cabo Rojo, PR, USA; a pine forest near Cabo Rojo, PR, USA; mangrove swamp in Carolina, PR, USA; Mayaguez, PR, USA; Cuzdrioara, Romania; Brazi Reservoir, Rosia Montana, Romania; Crow's Rock, Rosia Montana, Romania; pine tree forest, Rosia Montana, Romania; Yokohama City, Kanagawa Prefecture, Japan; beach in Kochin, India; river sediment from Kerala, India.

Supplementary References

1. Rice, E. W., Baird. R. B., Eaton, A. D., & Clessari, C. S. Standard methods for the examination of water and wastewaters. 22nd Ed. Washington DC, USA (2012).
2. Marsili, E., Rollefson, J. B., Baron, D. B., Hozalski, R. M., & Bond, D. R. Microbial Biofilm Voltammetry: Direct Electrochemical characterization of a catalytic electrode attached biofilms. *Appl Environ Microbiol* **74**, 7329 – 7337 (2008).
3. Jain, A., Gazzola, G., Panzera, A., Zanoni, M., & Marsili, E. Visible spectroelectrochemical characterization of *Geobacter sulfurreducens* biofilms on optically transparent indium tin oxide electrode. *Electrochim Acta* **56**, 10776 – 10785 (2011).
4. Richter, H. et al. Electricity generation by *Geobacter sulfurreducens* attached to gold electrodes. *Langmuir* **24**, 4376 – 4879 (2008).
5. Katuri, K. P., Kavanagh, P., Rengaraj, S., & Leech, D. *Geobacter sulfurreducens* biofilms developed under different growth conditions on glassy carbon electrodes: insights using cyclic voltammetry. *ChemComm* **46**, 4758 – 4760 (2010).
6. Parameswaran, P. et al. Kinetic, Electrochemical, and Microscopic characterization of thermophilic, anode-respiring bacterium *Thermincola ferriacetica. Environ Sci Technol* **47**, 4934 – 4940 (2013).
7. Sun, D., Call, D., Wang, A., Cheng, S., & Logan, B. E. *Geobacter* sp. SD-1 with enhanced electrochemical activity in high salt concentration solutions. *Environ Microbiol Rep* **6**, 723 – 729 (2014).

8. Carmona-Martinez, A. A. et al. Cyclic-Voltammetric analyses of the electron transfer of *Shewanella oneidensis* MR-1 and nanofilament and cytochrome knock-out mutants. *Bioelectrochemistry* **81**, 74 – 80 (2011).

9. Newton, G. J., Mori, S., Nakamura, R., Hashimoto, K., & Watanable, K. Analyses of the current generating mechanism of *Shewanella loihica* PV-4 and *Shewanella oneidensis* MR-1 in microbial fuel cells. *Appl Environ Microbiol* **75**, 7674 – 7681 (2009).

10. Wu, D. et al. Ferric iron enhance electricity generation by *Shewanella oneidensis* MR-1 in microbial fuel cells. *Bioresour Technol* **135**, 630 – 634 (2013).

11. Carmona-Martinez, A. A., Pierra, M., Trably, C., & Bernet, N. High current density *via* direct electron transfer by the halophilic anode respiring bacterium *Geoalkalibacter subterraneus. Phys Chem Chem Phys* **15**, 19699-19707 (2013).

12. Miceli, J. F., Parameswaran, P., Kang, D. W., Brown, R. K., & Torres, C. I. Enrichment and analysis of anode-respiring bacteria from diverse anaerobic inocula. *Environ Sci Technol* **46**, 10349 – 10355 (2012).

13. Abrevaya, X. C., Sacco, N., Mauas, P. J. D., & Corton, E. Archaea-base Microbial Fuel Cell operating at high ionic strength conditions. *Extremophiles* **15**, 633 – 642 (2011).

14. Badia-Fabregat, M., Rago, L., Baeza, J. A., & Guisasola, A. Hydrogen production from crude glycerol in an alkaline microbial electrolysis cell. *Int J Hydrogen Energ* **14**, 17004 – 17213 (2015).

15. Badalamenti, J. P., Brown, R. K., & Torres, C. I. Generation of high current densities by pure cultures of anode respiring *Geloalkalibacter* spp. under alkaline and saline conditions in Microbial Electrochemical Cells. *mBio* **4**, e00144-13 (2013).

16. Kumar, K. S., Feria, O. S., Ramirez, J. T., Seijas, N. R., & Varalso, H. M. P. Electrochemical and chemical enrichment of a sodic-saline inoculum for microbial fuel cells. *Int J Hydrogen Energy* **38**, 12600-12609 (2013).
17. Ledezma, P., Lu, Y., & Freguia, S. Electroactive haloalkaliphiles exhibit exceptional tolerance to free ammonia. *FEMS Microbiol Lett* **365**, fnx260 (2018).
