# Supplementary material for: Microbial electroactive biofilms dominated by Geoalkalibacter spp. from a highly saline–alkaline environment
Source: NPJ Biofilms Microbiomes. 2020 Oct 13;6:38. doi: 10.1038/s41522-020-00147-7 (PMC7555509; doi:10.1038/s41522-020-00147-7)
Supplement: Supplementary file 2 — Reporting Summary [file 41522_2020_147_MOESM2_ESM.pdf]

nature research

Corresponding author(s): Dr. Sunil A. Patil

Last updated by author(s): Sep 7, 2020

# Reporting Summary

Nature Research wishes to improve the reproducibility of the work that we publish. This form provides structure for consistency and transparency in reporting. For further information on Nature Research policies, see our Editorial Policies and the Editorial Policy Checklist.

## Statistics

For all statistical analyses, confirm that the following items are present in the figure legend, table legend, main text, or Methods section.

| n/a | Confirmed | |
|---|---|---|
| ☐ | ☒ | The exact sample size ($n$) for each experimental group/condition, given as a discrete number and unit of measurement |
| ☐ | ☒ | A statement on whether measurements were taken from distinct samples or whether the same sample was measured repeatedly |
| ☒ | ☐ | The statistical test(s) used AND whether they are one- or two-sided<br>*Only common tests should be described solely by name; describe more complex techniques in the Methods section.* |
| ☐ | ☒ | A description of all covariates tested |
| ☒ | ☐ | A description of any assumptions or corrections, such as tests of normality and adjustment for multiple comparisons |
| ☒ | ☐ | A full description of the statistical parameters including central tendency (e.g. means) or other basic estimates (e.g. regression coefficient) AND variation (e.g. standard deviation) or associated estimates of uncertainty (e.g. confidence intervals) |
| ☒ | ☐ | For null hypothesis testing, the test statistic (e.g. $F$, $t$, $r$) with confidence intervals, effect sizes, degrees of freedom and $P$ value noted<br>*Give P values as exact values whenever suitable.* |
| ☒ | ☐ | For Bayesian analysis, information on the choice of priors and Markov chain Monte Carlo settings |
| ☒ | ☐ | For hierarchical and complex designs, identification of the appropriate level for tests and full reporting of outcomes |
| ☒ | ☐ | Estimates of effect sizes (e.g. Cohen's $d$, Pearson's $r$), indicating how they were calculated |

*Our web collection on statistics for biologists contains articles on many of the points above.*

## Software and code

Policy information about availability of computer code

| | |
|---|---|
| Data collection | 1. EC-Lab electrochemical software pre-installed with the potentiostat/galvanostat was used to collect the electrochemical data.<br>2. Computing work station with Linux operating system was used to relate and analyze the metagenome sequencing data.<br>3. JEOL IT-100 TouchScope software was used to visualize the biofilm coverage over the anodes. |
| Data analysis | 1. Origin Pro software was used to analyze and plot all the electrochemical data.<br>2. R package, Microsoft excel and Origin were used to analyze and plot the metagenome sequencing data.<br>3. Fiji-Image J software was used to process the microscopic images. |

For manuscripts utilizing custom algorithms or software that are central to the research but not yet described in published literature, software must be made available to editors and reviewers. We strongly encourage code deposition in a community repository (e.g. GitHub). See the Nature Research guidelines for submitting code & software for further information.

## Data

Policy information about availability of data

All manuscripts must include a data availability statement. This statement should provide the following information, where applicable:

- Accession codes, unique identifiers, or web links for publicly available datasets
- A list of figures that have associated raw data
- A description of any restrictions on data availability

All data generated or analyzed during this study are included in this article and its Supplementary Information file. All raw sequencing data are available on the NCBI archive with the project accession number PRJNA604728.

# Field-specific reporting

Please select the one below that is the best fit for your research. If you are not sure, read the appropriate sections before making your selection.

☐ Life sciences ☐ Behavioural & social sciences ☒ Ecological, evolutionary & environmental sciences

For a reference copy of the document with all sections, see nature.com/documents/nr-reporting-summary-flat.pdf

# Ecological, evolutionary & environmental sciences study design

All studies must disclose on these points even when the disclosure is negative.

| | |
|---|---|
| Study description | This study aimed at understanding of the microbial electroactive biofilms (EABs) possessing the outward extracellular electron transfer (EET) capabilities from the highly saline-alkaline environment. We used the electrochemical cultivation approach to enrich haloalkaliphilic EABs under 9.5 pH and 20 g/L salinity conditions and different substrate conditions. The electrodes were used as the proxy to the (solid-state) terminal electron acceptors. The enriched EABs were characterized using electrochemical, microscopic and metagenomics approaches. The electrodes controlled at 0.2 V vs. Ag/AgCl yielded the best-performing biofilms in terms of maximum bioelectrocatalytic current densities of 548±23 and 437±17 µA/cm2 with acetate and lactate substrates, respectively. Electrochemical characterization of biofilms revealed the presence of two novel putative redox-active moieties with the mean formal potentials of 0.183 and 0.333 V vs. Ag/AgCl, which represent the highest values reported to date for the EABs. Metagenomics of EABs revealed the dominance of novel Geoalkalibacter sp. at about 80% abundance. The novel haloalkaliphilic EABs possessing EET components with high formal potentials reported in this study offers exciting research prospects in extreme electromicrobiology. |
| Research sample | At least three peripheral locations of the Lonar Lake were selected for sampling to minimize uncertainties in the physicochemical characteristics of the sediment samples and to get the representative microbial inoculum source from the lake system. All microbial cultivation experiments via electrochemical approach were conducted in at least triplicates. |
| Sampling strategy | Sediment samples from up to a depth of ~1 ft. from the surface were collected in air-tight amber-colored sampling bottles of 1 L capacity. |
| Data collection | The electrochemical data was collected using the potentiostat equipped with the EC-Lab software of the Biologic Science Instruments, France. The specific techniques used include chronoamperometry and cyclic voltammetry. The sediment characterization was done according to the standard protocols of APHA. The SEM images of the microbial biofilms were acquired using JEOL JSM-6010PLUS/LS scanning electron microscope. Metagenome amplicon sequencing for 16S rRNA (V3-V4) regions was conducted at Illumina HiSeq template. The obtained raw sequences were preprocessed using various bioinformatic tools viz. PERL, FLASH, UCHIME-V11 and VSEARCH. QIIME software was used to align taxonomy and check the relative OTU abundance within the preprocessed metagenome sequences based on the similarity alignment with SILVA database. |
| Timing and spatial scale | Sediment sampling was done during the morning in between 6 to 11 am. The electric current data was collected after each 2 minutes throughout the operation and cyclic voltammetry was recorded at scan rate of 1 mV/s. |
| Data exclusions | Particularly for metagenome sequencing data, the microbial communities with <1 % were reported as others. So, we have excluded that only. |
| Reproducibility | All the experiments and analyses were conducted in at least triplicates to check for the reproducibility of the data. |
| Randomization | The sediment sampling sites were were selected randomly. |
| Blinding | Blinding was not possible for this study. Because whole the data viz. electrochemical and metagenomic were collected and analyzed using EC-Lab and illumina sequencing compared with bacterial/Archaeal library of SILVA database. All these tools are very well developed and are being considered as the best tools with very little blinding rate. |

Did the study involve field work? ☒ Yes ☐ No

## Field work, collection and transport

| | |
|---|---|
| Field conditions | Temperature 25±2, pH 10 |
| Location | Lonar Lake, Buldhana District, Maharashtra, India. Coordinates 19°58′35″N 76°30′30″E |
| Access & import/export | N/A |
| Disturbance | No disturbance was caused during the sampling. |

# Reporting for specific materials, systems and methods

We require information from authors about some types of materials, experimental systems and methods used in many studies. Here, indicate whether each material, system or method listed is relevant to your study. If you are not sure if a list item applies to your research, read the appropriate section before selecting a response.

## Materials & experimental systems

| n/a | Involved in the study |
| --- | --- |
| ☒ | ☐ Antibodies |
| ☒ | ☐ Eukaryotic cell lines |
| ☒ | ☐ Palaeontology and archaeology |
| ☒ | ☐ Animals and other organisms |
| ☒ | ☐ Human research participants |
| ☒ | ☐ Clinical data |
| ☒ | ☐ Dual use research of concern |

## Methods

| n/a | Involved in the study |
| --- | --- |
| ☒ | ☐ ChIP-seq |
| ☒ | ☐ Flow cytometry |
| ☒ | ☐ MRI-based neuroimaging |
